# Klotho inhibits IGF1R/PI3K/AKT signalling pathway and protects the heart from oxidative stress during ischemia/reperfusion injury

**DOI:** 10.1038/s41598-023-47686-5

**Published:** 2023-11-20

**Authors:** Agnieszka Olejnik, Anna Radajewska, Anna Krzywonos-Zawadzka, Iwona Bil-Lula

**Affiliations:** Division of Clinical Chemistry and Laboratory Haematology, Department of Medical Laboratory Diagnostics, Faculty of Pharmacy, Wroclaw Medical University, Borowska 211A St., 50-556 Wrocław, Poland

**Keywords:** Cell signalling, Cardiovascular biology

## Abstract

Ischemia/reperfusion injury (IRI) of the heart involves the activation of oxidative and proapoptotic pathways. Simultaneously Klotho protein presents anti-aging, antiapoptotic and antioxidative properties. Therefore, this study aimed to evaluate the effect of Klotho protein on oxidative stress in hearts subjected to IRI. Isolated rat hearts perfused with the Langendorff method were subjected to ischemia, followed by reperfusion, in the presence or absence of recombinant rat Klotho protein. The factors involved in the activation of insulin-like growth factor receptor (IGF1R)/phosphoinositide-3-kinase (PI3K)/protein kinase B (AKT) signalling pathway were evaluated. IRI caused activation of the IGF1R (*p* = 0.0122)/PI3K (*p* = 0.0022) signalling, as compared to the aerobic control group. Infusion supply of Klotho protein during IRI significantly reduced the level of phospho-IGF1R (*p* = 0.0436), PI3K (*p* = 0.0218) and phospho-AKT (*p* = 0.0020). Transcriptional activity of forkhead box protein O3 (FOXO3) was reduced (*p* = 0.0207) in hearts subjected to IRI, compared to aerobic control. Administration of Klotho decreased phosphorylation of FOXO3 (*p* = 0.0355), and enhanced activity of glutathione peroxidase (*p* = 0.0452) and superoxide dismutase (*p* = 0.0060) in IRI + Klotho group. The levels of reactive oxygen/nitrogen species (ROS/RNS) (*p* = 0.0480) and hydrogen peroxide (H_2_O_2_) (*p* = 0.0460), and heart injury (*p* = 0.0005) were significantly increased in hearts from the IRI group in comparison to the aerobic group. Klotho reduced NADPH oxidase 2 (NOX2) (*p* = 0.0390), ROS/RNS (*p* = 0.0435) and H_2_O_2_ (*p* = 0.0392) levels, and heart damage (*p* = 0.0286) in the hearts subjected to IRI. In conclusion, Klotho contributed to the protection of the heart against IRI and oxidative stress via inhibition of the IGF1R/PI3K/AKT pathway, thus can be recognized as a novel cardiopreventive/cardioprotective agent.

## Introduction

Deficient blood supply to tissues due to obstruction of the arterial inflow, and subsequent restoration of perfusion can cause ischemia/reperfusion injury (IRI)^1^. While ischemia results in cell dysfunction, injury and/or death, reperfusion can enhance the damage that occurred. Moreover, reperfusion is related to release of mediators that involve morphological, metabolic and contractile disorders in distant organs^1^. One of the major pathological events contributing to IRI is the excessive formation of reactive oxygen/nitrogen species (ROS/RNS) and oxidative stress^2^. As a result of oxidative stress, oxidative or nitrosative modification of key regulatory proteins and alteration of cell signalling occur^1^. An important ROS/RNS produced during IRI are superoxide (O_2_^·−^) and nitric oxide (NO^·^). They react with each other, and with other radical and non-radical (oxidants) entities, and form a diverse array of additional ROS and RNS^3^. A significant source of ROS is the electron transport chain in mitochondria, as well as enzymes like membrane nicotinamide adenine dinucleotide phosphate (NADPH) oxidases (NOXs), and cytosolic xanthine and xanthine oxidase^1,3,4^.

Forkhead box protein O (FOXO) transcription factors play an important role in cell proliferation, apoptosis, autophagy, metabolism, inflammation and differentiation by activation or inhibition of downstream target genes^5^. Moreover, FOXOs are related to resistance to oxidative stress and work as a negative regulator of mitochondrial ROS production^6^. The most widely studied members of the FOXO subfamily are FOXO1 and FOXO3. It was shown that FOXO1 reduced oxidative stress and apoptosis of hematopoietic stem cells, pancreatic ß-cells, or keratinocytes. Then, FOXO1 and FOXO3 induced autophagy and reduced cardiac hypertrophy after IRI^5^. Importantly, FOXO3 plays a role in oxidative stress protection by increasing the production of antioxidants like manganese superoxide dismutase (MnSOD), catalase, or peroxiredoxin III^5^.

The subcellular location, DNA binding affinity, and transcriptional activity of FOXOs are modified by their phosphorylation by several protein kinases, e.g. serine-threonine protein kinase B (AKT)^5^. It is known that FOXOs are key transcriptional targets of insulin/insulin-like growth factor 1 (IGF1)/phosphoinositide-3-kinase (PI3K) signalling pathway^5,7^. The activation of insulin-like growth factor receptor (IGF1R) results in phosphorylation and activation of PI3K and AKT. Then, AKT phosphorylates FOXO^8^. As a result of phosphorylation of FOXO, the translocation of FOXO from the nucleus to the cytoplasm is observed. It leads to accumulation of phosphorylated FOXO in the cytosol and its transcriptional inactivation^5^.

Klotho is a membrane-bound or soluble antiaging protein, and is expressed mainly in the kidneys and the brain. It is a coreceptor for fibroblast growth factor (FGF) and hence regulates endocrine system homeostasis^9^. Since Klotho supports redox balance and metabolic functions of the cardiomyocytes, it was proposed as a component in the therapeutic strategy for aging-associated cardiomyopathy and heart failure^10,11^. The same study showed that Klotho may be a novel biomarker for responsiveness against treatment in patients with heart failure^12^. However, further studies are needed to explore the underlying molecular mechanism of Klotho activity and cardioprotection that follows.

This study aimed to evaluate the effect of Klotho protein on oxidative stress in terms of the IGF1R/PI3K/FOXO3 signalling pathway in the heart subjected to IRI.

## Materials and methods

### Heart tissue homogenates

In this study, remaining cardiac tissue homogenates obtained under the National Science Centre Poland project number 2019/33/N/NZ3/01649 approved by the Ethics Committee for Experiments on Animals at the Ludwik Hirszfeld Institute of Immunology and Experimental Therapy Polish Academy of Sciences, Wroclaw, Poland (Resolution 002/2020 of 15th January 2020) were used. All experimental procedures in the animals were performed following the published Guide of the Polish Ministry of Science and Higher Education for the Care and Use of Experimental Animals. The study was performed in accordance with the ARRIVE (Animal Research: Reporting of In Vivo Experiments) guidelines 2.0. The heart tissue used in this project was prepared under the procedures carried out in project number 2019/33/N/NZ3/01649 as follows^13^:

#### Isolation of rat hearts and perfusion with the Langendorff method

Adult male Wistar rats weighing 200–350 g were obtained from Mossakowski Medical Research Center, Polish Academy of Sciences, Warsaw, Poland. The animals were housed in cages (two rats/cage) and kept at a controlled temperature (22 ± 2 °C), humidity (55 ± 5%), and light/dark (12/12 h) cycle. An ad libitum access to a diet of standard laboratory chow and water was provided. Rats were desensitized with buprenorphine (0.05 mg/kg, *i. p*.), anesthetized with sodium pentobarbital (0.5 ml/kg *i. p.*), and the hearts were rapidly excised from animals. The spontaneously beating hearts were then rinsed by immersion in ice‐cold Krebs‐Henseleit Buffer (118 mmol/L NaCl, 4.7 mmol/L KCl, 1.2 mmol/L KH_2_PO_4_, 1.2 mmol/L MgSO_4_, 3.0 mmol/L CaCl_2_, 25 mmol/L NaHCO_3_, 11 mmol/L glucose, and 0.5 mmol/L EDTA, pH 7.4) (Sigma‐Aldrich, Munich, Germany), and cannulated by the aorta on the Langendorff system (EMKA Technologies, Paris, France). The above procedure was completed within 30 s. Then, hearts were perfused at constant pressure (60 mmHg) with Krebs–Henseleit Buffer (pH 7.4, 37 °C) and gassed continuously (5% CO_2_/95% O_2_).

#### Global ischaemia/reperfusion injury of isolated rat hearts

**Figure 1:**
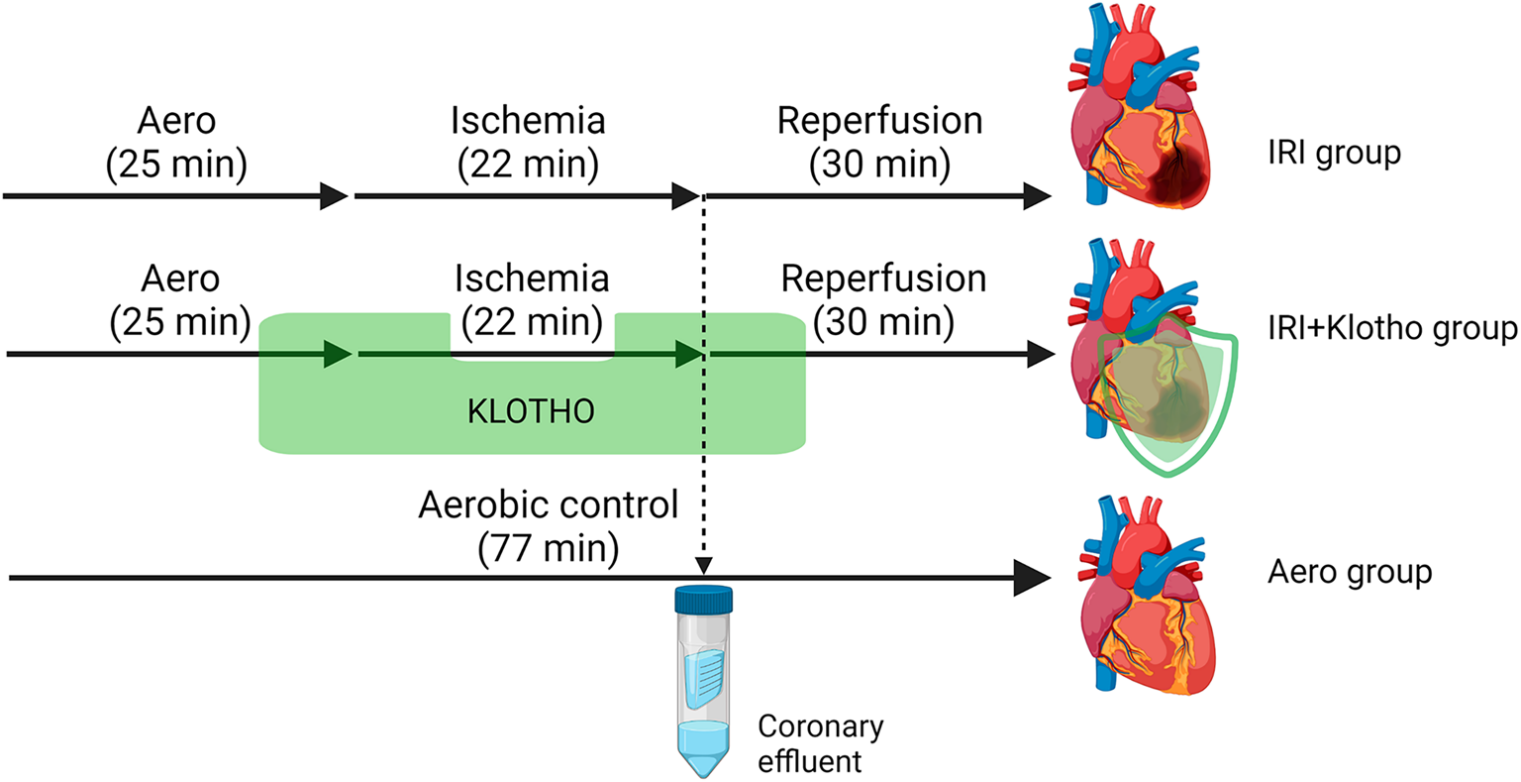
Experimental protocol of heart IRI. IRI—ischemia/reperfusion injury. Figure created with BioRender (https://biorender.com/).

Rat hearts were distributed randomly into 3 groups: aerobic control without Klotho (aero group, N = 9), acute myocardial IRI without Klotho (IRI group, N = 14), and acute myocardial IRI with Klotho (IRI + Klotho group, N = 7). The scheme of the experimental protocol of the heart IRI is shown in Fig. 1. The isolated hearts from the IRI groups underwent 25 min of aerobic stabilization, 22 min of global no‐flow ischaemia (by a cessation of the buffer flow), and 30 min of reperfusion (aerobic conditions) in the presence or absence of Klotho protein. The hearts from the aerobic group were perfused aerobically for 77 min. Recombinant rat αKlotho protein (RPH757Ra01, Cloud-Clone Corp., USA) was diluted to a final concentration of 0.5 ng/mL with Krebs–Henseleit buffer, immediately before administration. The optimal concentration of Klotho protein was determined experimentally^13^. Klotho was administered with the perfusion buffer into the hearts during the last 10 min of aerobic stabilization and in the first 10 min of reperfusion (after global ischemia) (Fig. 1). After the experimental protocol, isolated hearts were immediately immersed in liquid nitrogen and stored at − 80 °C for further investigations. 15 mL of coronary effluents were collected at the beginning of reperfusion (47 min of the experiment) (Fig. 1). Then, coronary effluents were concentrated (1 mL final volume) using Amicon Ultra-15 Centrifugal Filter Units with Ultracel-10 membrane (EMD Millipore, USA), aliquoted, and frozen at − 80 °C for further biochemical analysis.

#### Preparation of heart homogenates

Frozen hearts were crushed using a mortar and pestle in liquid nitrogen. Then, heart tissue was homogenized mechanically in ice‐cold homogenization buffer (50 mmol/L Tris‐HCl (pH 7.4), 3.1 mmol/L sucrose, 1 mmol/L dithiothreitol, 10 mg/mL, leupeptin, 10 mg/mL soybean trypsin inhibitor, 2 mg/mL aprotinin and 0.1% Triton X‐100) (Sigma‐Aldrich, Munich, Germany). The homogenate was centrifuged (10 000 × g at 4 °C for 15 min), and the supernatant was collected and stored at − 80 °C for further biochemical experiments. Exactly the same initial tissue mass and the corresponding fourfold volume of homogenization buffer were used to homogenize each heart. Each heart was homogenized using exactly the same procedure to normalize the amount of tissue and protein in each final sample. The obtained supernatants did not differ statistically in the protein concentration in each study group (data not shown).

### IGF1R and p-IGF1R levels

The level of total IGF1R in the heart tissue was determined using Rat insulin-like growth factor 1 receptor (IGF1R) ELISA Kit (Wuhan Newqidi Biotech, Wuhan, China). Briefly, primary capture antibodies bind IGF1R from the sample. Then, IGF1R is detected with biotinylated anti-IGF1R secondary antibodies and streptavidin–horseradish peroxidase (HRP)-avidin complex. The substrate solution is then added, and colour develops in proportion to the amount of total rat IGF1R.

To verify pathway activation, phospho-IGF1R (p-IGF1R) level in heart homogenates using Rat phospho-insulin-like growth factor 1 receptor (p-IGF1R) ELISA Kit (Wuhan Newqidi Biotech, Wuhan, China) was measured. Briefly, p-IGFR is tied with antibody specific to rat p-IGF1R, and is detected by biotin-conjugated polyclonal antibody and HRP-avidin. Next, 3,3′,5,5′ Tetramethylbenzidine (TMB) substrate is used to enable visualization of the reaction and colour develops in proportion to the amount of rat p-IGF1R.

### PI3K level

The level of PI3K in heart tissue was evaluated with Rat Phosphotylinosital 3 Kinase (PI3K) ELISA Kit (Cusabio, Houston, USA). Briefly, PI3K present in the sample is bound by the specific immobilized antibody. Then, a biotin-conjugated antibody specific for PI3K and HRP-avidin are added. A substrate solution is added to the wells, and color develops in proportion to the amount of PI3K bound.

### AKT and p-AKT levels

To quantitatively measure total AKT in the heart homogenates, RayBio® Human/Mouse/Rat Total AKT ELISA Kit (RayBiotech Life, Peachtree Corners, USA) according to manufacturer’s instruction was used. Briefly, AKT present in a sample is bound by the immobilized antibody and rabbit anti-pan-Akt antibody is used to detect total AKT.

To monitor the activation of AKT, the level of AKT phosphorylated at S473 in the heart tissue using the Rat Phospho-AKT(S473) ELISA Kit (BT-Laboratory, Zhejiang, China) according to manufacturer’s instruction was measured.

### FOXO3 and p-FOXO3 levels

Rat Forkhead Box Protein O3 (FOXO3) ELISA Kit (Wuhan Newqidi Biotech, Wuhan, China) for quantitative detection of total rat FOXO3 in the heart tissue was used. The kit utilizes the “Double Antibody Sandwich” ELISA technique. The pre-coated antibody is an anti-Rat FOXO3 monoclonal antibody, while the detection antibody is a biotinylated anti-Rat FOXO3 polyclonal antibody. The color intensity and quantity of total FOXO3 in the sample were positively correlated. Then, Rat phospho Forkhead Box Protein O3 (p-FOXO3) ELISA Kit (Wuhan Newqidi Biotech, Wuhan, China) according to manufacturer’s instruction was used for evaluation of p-FOXO3 level in the heart tissue.

### Superoxide dismutase activity

SOD Assay Kit (Sigma‐Aldrich, Munich, Germany) was used for the determination of superoxide dismutase activity in the heart tissue. Briefly, the highly water-soluble tetrazolium salt is reduced with a superoxide anion and produces a water-soluble formazan dye. The rate of the reduction with O_2_^·−^ is linearly related to the xanthine oxidase activity, and is inhibited by SOD. The absorbance is proportional to the amount of O_2_^·−^. The SOD activity, as an inhibition activity, can be quantified by measuring the decrease in colour.

### Glutathione peroxidase activity

Glutathione peroxidase (GPx) activity in the heart tissue was determined using the Glutathione Peroxidase Assay Kit (Cayman Chemical, Michigan USA). Briefly, oxidized glutathione, produced upon reduction of hydroperoxide by GP_x_, is recycled to its reduced state by glutathione reductase and NADPH. The oxidation of NADPH to NADP^+^ is accompanied by a decrease in absorbance, which is proportional to the GP_x_ activity in the sample.

### NOX2 level

The level of NOX2 was assessed with the Rat Nicotinamide Adenine Dinucleotide Phosphate Oxidase 2 (NOX2) ELISA Kit (Wuhan Newqidi Biotech, Wuhan, China), based on the “Double Antibody Sandwich” technique. NOX2 level was evaluated in the heart tissue, according to manufacturer’s instruction.

### Assessment of oxidative stress

An OxiSelect™ In Vitro ROS/RNS Assay Kit (Cell Biolabs, San Diego, USA) was used to assess the levels of total ROS/RNS and hydrogen peroxide (H_2_O_2_) in the heart tissue. The kit measures total ROS/RNS or H_2_O_2_ with the fluorogenic probe—dichlorodihydrofluorescein (DCFH) DiOxyQ. If the sample contains ROS and RNS, the DCFH is rapidly oxidized to the highly fluorescent 2′,7′-dichlorodihydrofluorescein. The DCFH-DiOxyQ probe reacts with peroxyl radical (ROO·), NO^·^, and peroxynitrite, which are representative of both ROS and RNS. The level of H_2_O_2_ was assessed using the H_2_O_2_ standard and adequate standard curve. Fluorescence intensity was proportional to the total ROS/RNS or H_2_O_2_ levels within the sample.

### LDH activity

Lactate dehydrogenase (LDH) activity served as a marker of heart injury. The activity of LDH was assessed with the Lactate Dehydrogenase Activity Assay Kit (Sigma‐Aldrich, Munich, Germany) according to the manufacturer’s instruction. LDH is a stable cytosolic enzyme that is released into extracellular space upon cell membrane damage/permeability or cell lysis. Briefly, LDH interconverts pyruvate and lactate with the reduction of NAD to NADH, which is detected with a colorimetric assay at 450 nm. LDH activity was determined in coronary effluents.

### Statistical analysis

The data were analysed with GraphPad Prism 9 software (GraphPad Software, San Diego, CA, USA). To assess the normality of variance changes, the Shapiro‐Wilk normality test was used, and in all the variables *p* < 0.05 was considered significant. The equality of group variances with the Brown-Forsythe test was assessed. The comparison of data between groups was made with ANOVA, Welch’s ANOVA or nonparametric Kruskal–Wallis test with the post hoc tests (Tukey's or Dunn's multiple comparisons tests). The correlation analysis was assessed with Pearson’s or Spearman’s tests. Results were expressed as mean ± SD or box-and-whisker plots, with a value of *p* < 0.05 being regarded as statistically significant.

### Ethics approval

The manuscript does not contain clinical studies or patient data.

## Results

### Activation of IGF1R in heart tissue

**Figure 2:**
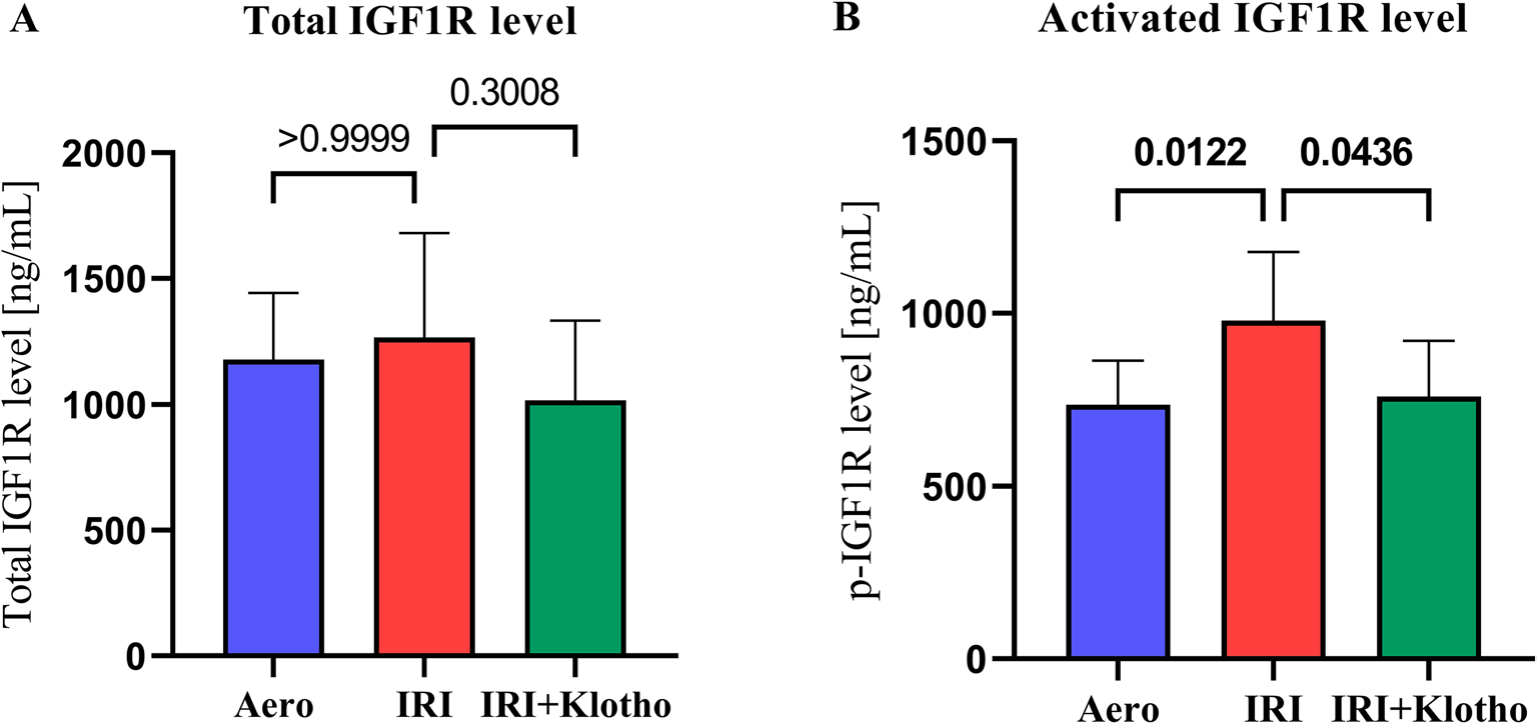
Activation of IGF1R in heart tissue. (**A**) The level of total IGF1R in the heart tissue; (**B**) The level of activated IGF1R; IGF1R—insulin-like growth factor 1 receptor; p-IGF1R—phosphorylated insulin-like growth factor 1 receptor; n_aero_ = 8–9; n_IRI_ = 13; n_IRI+Klotho_ = 6–7; mean ± SD.

There was no significant difference in the level of total IGF1R in the heart tissue between the groups (Fig. 2A). The level of activated IGF1R (p-IGF1R) was significantly increased in the IRI group compared to the aerobic control (Fig. 2B). Klotho significantly inhibited activation of IGF1R in the heart tissue during IRI (Fig. 2B).

### PI3K and AKT levels in rat hearts

**Figure 3:**
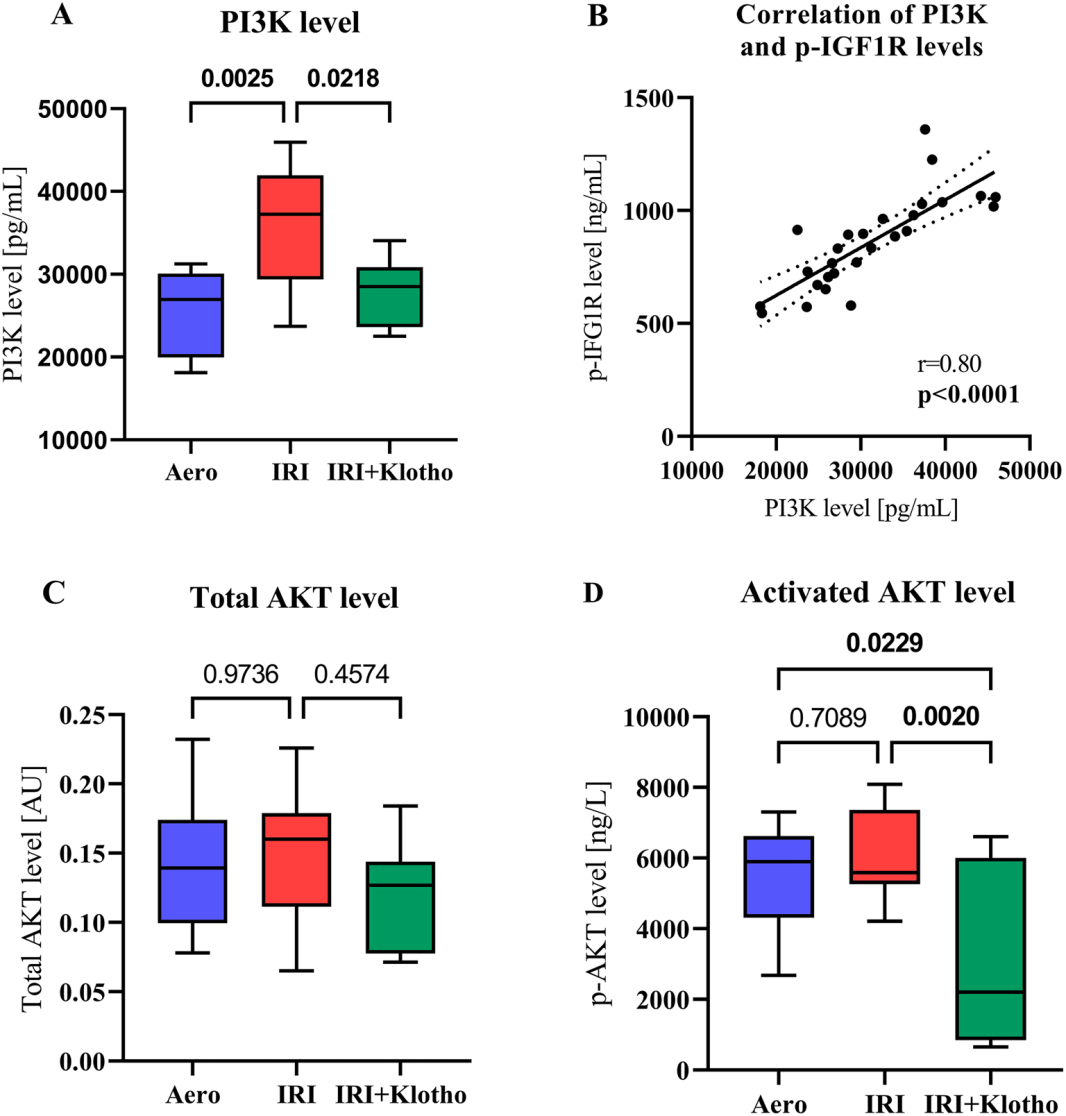
PI3K and AKT levels in rat hearts. (**A**) PI3K level in the heart tissue; (**B**) Correlation of PI3K and p-IGF1R levels; (**C**) Total AKT level; (**D**) Activation of AKT expressed as the level of p-AKT; AKT—protein kinase B; IGF1R—insulin-like growth factor 1 receptor; p-AKT—phosphorylated protein kinase B; p-IGF1R—phosphorylated insulin-like growth factor 1 receptor; PI3K—phosphoinositide-3-kinase; n_aero_ = 8; n_IRI_ = 12–14; n_IRI+Klotho_ = 7; boxes—25–75% percentile, whiskers—min to max + median.

The level of PI3K was significantly increased in the hearts after IRI as compared to aerobic group (Fig. 3A). There was a reduced level of PI3K in the IRI + Klotho group in comparison to the IRI group (Fig. 3A). PI3K level was positively correlated with p-IGF1R level (r = 0.80; *p* < 0.0001) (Fig. 3B).

The level of total AKT in the heart tissue did not show significant differences in the study groups (Fig. 3C). Administration of Klotho protein resulted in reduced activity of AKT in the hearts subjected to IRI (Fig. 3D).

### Inactivation of FOXO3

**Figure 4:**
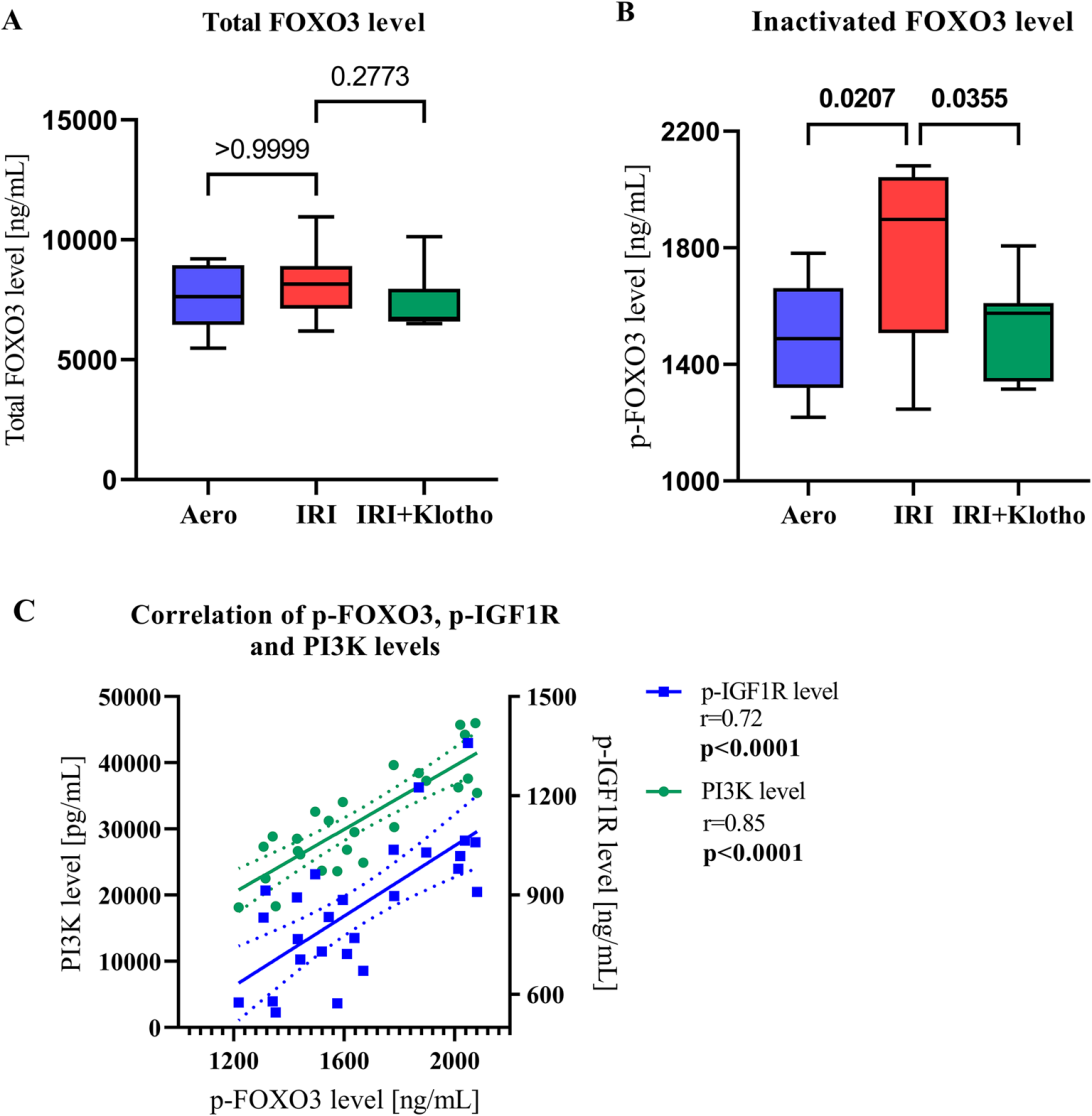
Inactivation of FOXO3. (**A**) The level of total FOXO3 in the heart tissue; (**B**) Inactivated FOXO3 level expressed as p-FOXO3; (**C**) Correlation of p-FOXO3 level with p-IGF1R and PI3K levels; FOXO3—forkhead box protein O 3; p-FOXO3—phosphorylated forkhead box protein O 3; p-IGF1R—phosphorylated insulin-like growth factor 1 receptor; PI3K—phosphoinositide-3-kinase; n_aero_ = 8–9; n_IRI_ = 13–14; n_IRI+Klotho_ = 7; boxes—25–75% percentile, whiskers—min to max + median.

There was no significant difference in the level of total FOXO3 in the heart tissue between the groups (Fig. 4A). IRI resulted in phosphorylation hence inactivation of FOXO3 when compared to the aerobic control (Fig. 4B). The level of inactivated FOXO3 was significantly reduced in the IRI + Klotho group (Fig. 4B). The level of inactivated FOXO3 was positively correlated with p-IGFR1 level (r = 0.72; *p* < 0.0001) (Fig. 4C) and PI3K level (r = 0.85; *p* < 0.0001) (Fig. 4C).

### Production of antioxidants in rat hearts

**Figure 5:**
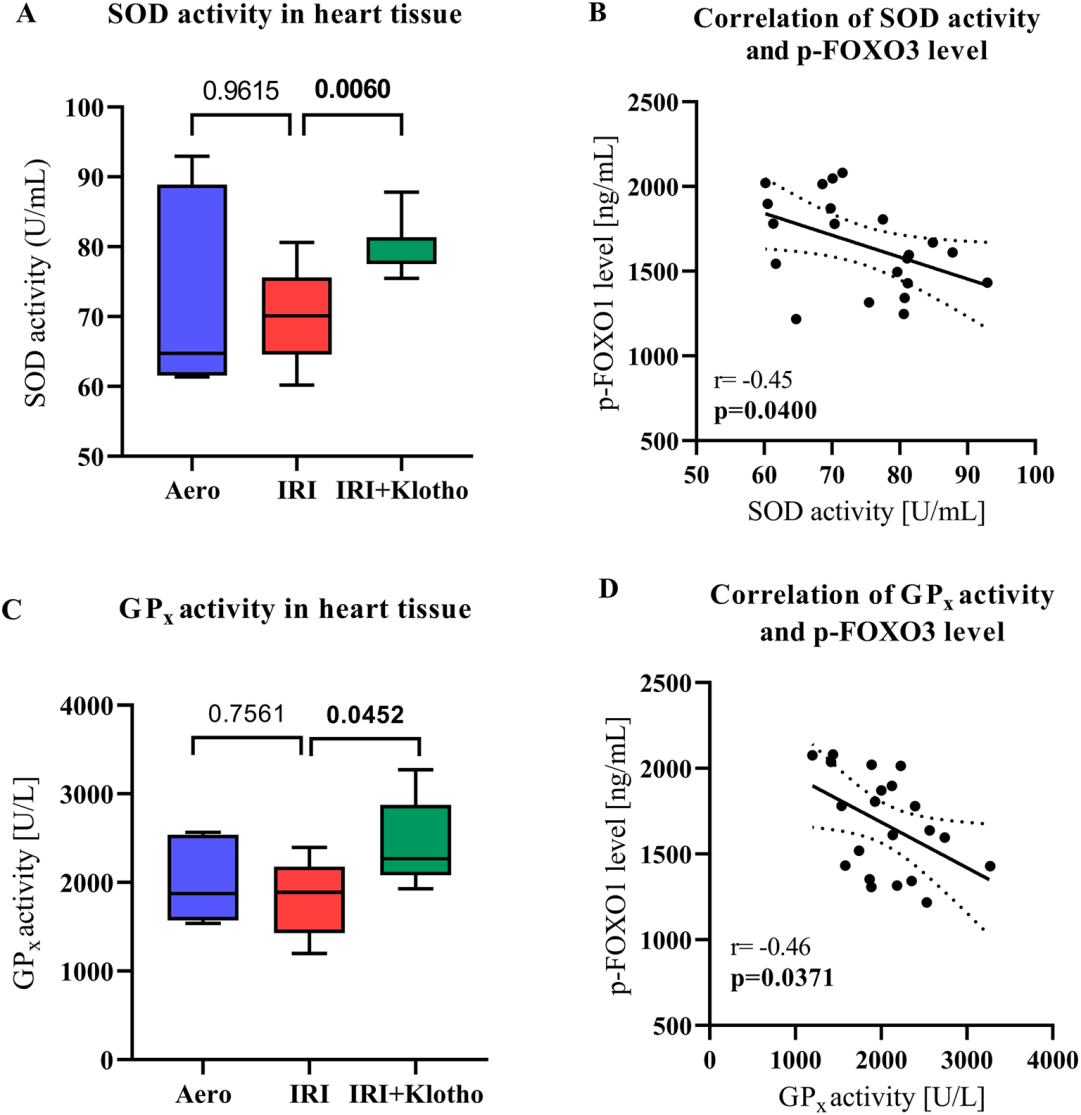
Production of antioxidants in rat hearts. (**A**) The activity of SOD in the heart tissue; (**B**) Correlation of SOD activity and p-FOXO3 level; (**C**) GP_X_ activity in the heart tissue; (**D**) Correlation of GP_X_ activity and p-FOXO3 level; GP_X_—glutathione peroxidase; p-FOXO3—phosphorylated forkhead box protein O 3; SOD—superoxide dismutase; n_aero_ = 5–6; n_IRI_ = 9; n_IRI+Klotho_ = 6–7; boxes—25–75% percentile, whiskers—min to max + median.

The activity of SOD was significantly increased in the IRI + Klotho group compared to the IRI group (Fig. 5A). There was a moderate negative correlation between SOD activity and p-FOXO3 level (r = − 0.45; *p* = 0.0400) (Fig. 5B).

The activity of GP_X_ was significantly increased in the hearts perfused with Klotho protein during IRI (Fig. 5C). There was a moderate negative correlation between GP_x_ activity and p-FOXO3 level (r = − 0.46; *p* = 0.0371) (Fig. 5D).

### NOX2 level in heart tissue

**Figure 6:**
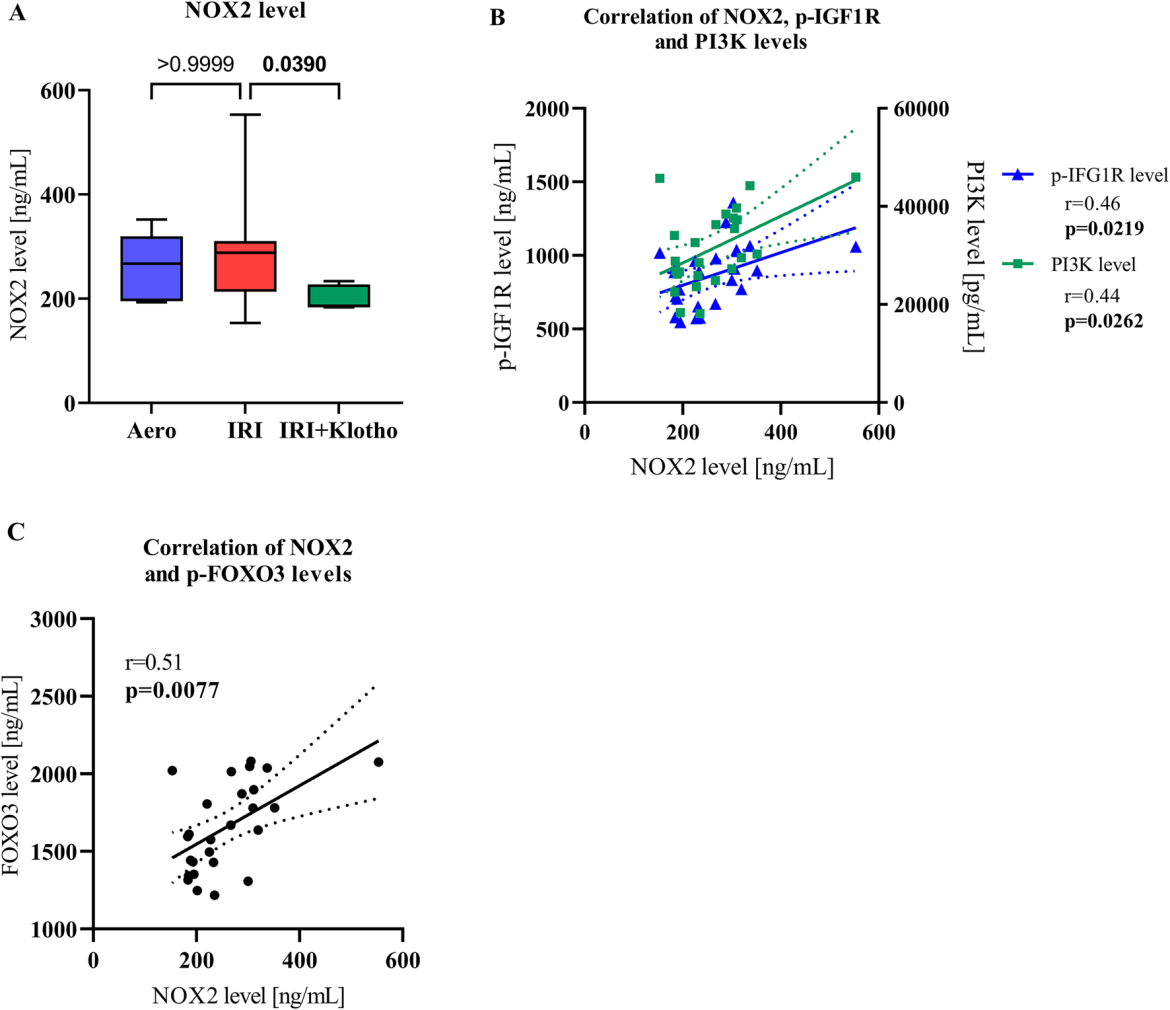
NOX2 level in heart tissue. (**A**) The level of NOX2 in the heart tissue; (**B**) Correlation of p-IGF1R, PI3K and NOX2 levels; (**C**) Correlation of FOXO3 and NOX2 levels; NOX2—nicotinamide adenine dinucleotide phosphate oxidase 2; p-FOXO3—phosphorylated forkhead box protein O 3; p-IGF1R—phosphorylated insulin-like growth factor 1 receptor; PI3K—phosphoinositide-3-kinase; n_aero_ = 7; n_IRI_ = 13; n_IRI+Klotho_ = 7; boxes—25–75% percentile, whiskers—min to max + median.

Perfusion of the hearts with Klotho protein during IRI contributed to the reduced level of NOX2 (Fig. 6A). There was a moderate correlation of NOX2 level with p-IGF1R level (r = 0.46; *p* = 0.0219) (Fig. 6B) and PI3K level (r = 0.44; *p* = 0.0262) (Fig. 6B). NOX2 level positively correlated with p-FOXO3 level (r = 0.51; *p* = 0.0077) (Fig. 6C).

### Magnitude of oxidative stress

**Figure 7:**
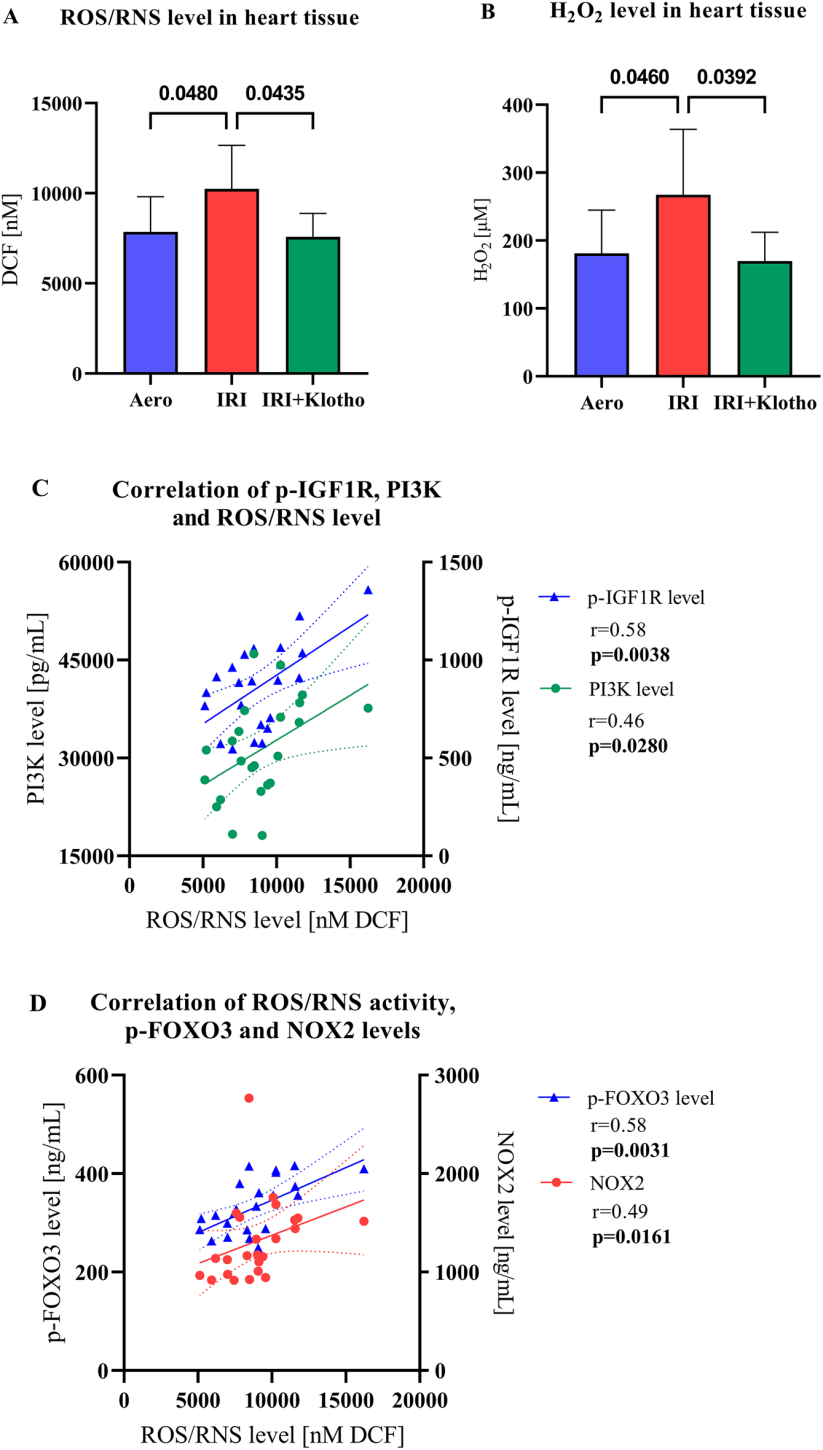
The magnitude of oxidative stress. (**A**) The level of ROS/RNS in the heart tissue; (**B**) The level of H_2_O_2_ in the heart tissue; (**C**) Correlation of ROS/RNS, p-IGF1R level and PI3K levels; (**D**) Correlation of ROS/RNS, p-FOXO3 and NOX2 levels; DCF—dichlorodihydrofluorescein; H_2_O_2_—hydrogen peroxide; NOX2—nicotinamide adenine dinucleotide phosphate oxidase 2; p-FOXO3—phosphorylated forkhead box protein O 3; p-IGF1R—phosphorylated insulin-like growth factor 1 receptor; PI3K—phosphoinositide-3-kinase; ROS/RNS—reactive oxygen/nitrogen species; n_aero_ = 8; n_IRI_ = 12; n_IRI+Klotho_ = 6; mean ± SD.

The levels of ROS/RNS (Fig. 7A) and H_2_O_2_ (Fig. 7B) were significantly increased in the hearts from the IRI group in comparison to the aero group. Klotho contributed to the reduction in ROS/RNS (Fig. 7A) and H_2_O_2_ (Fig. 7B) levels in the hearts subjected to IRI. The magnitude of oxidative stress was positively correlated with p-IGF1R level (r = 0.58; *p* = 0.0038) (Fig. 7C), PI3K level (r = 0.46; *p* = 0.0280) (Fig. 7C), p-FOXO3 level (r = 0.58; *p* = 0.0031) (Fig. 7D), and with NOX2 level (r = 0.49; *p* = 0.0161) (Fig. 7D).

### Intensity of heart injury

**Figure 8:**
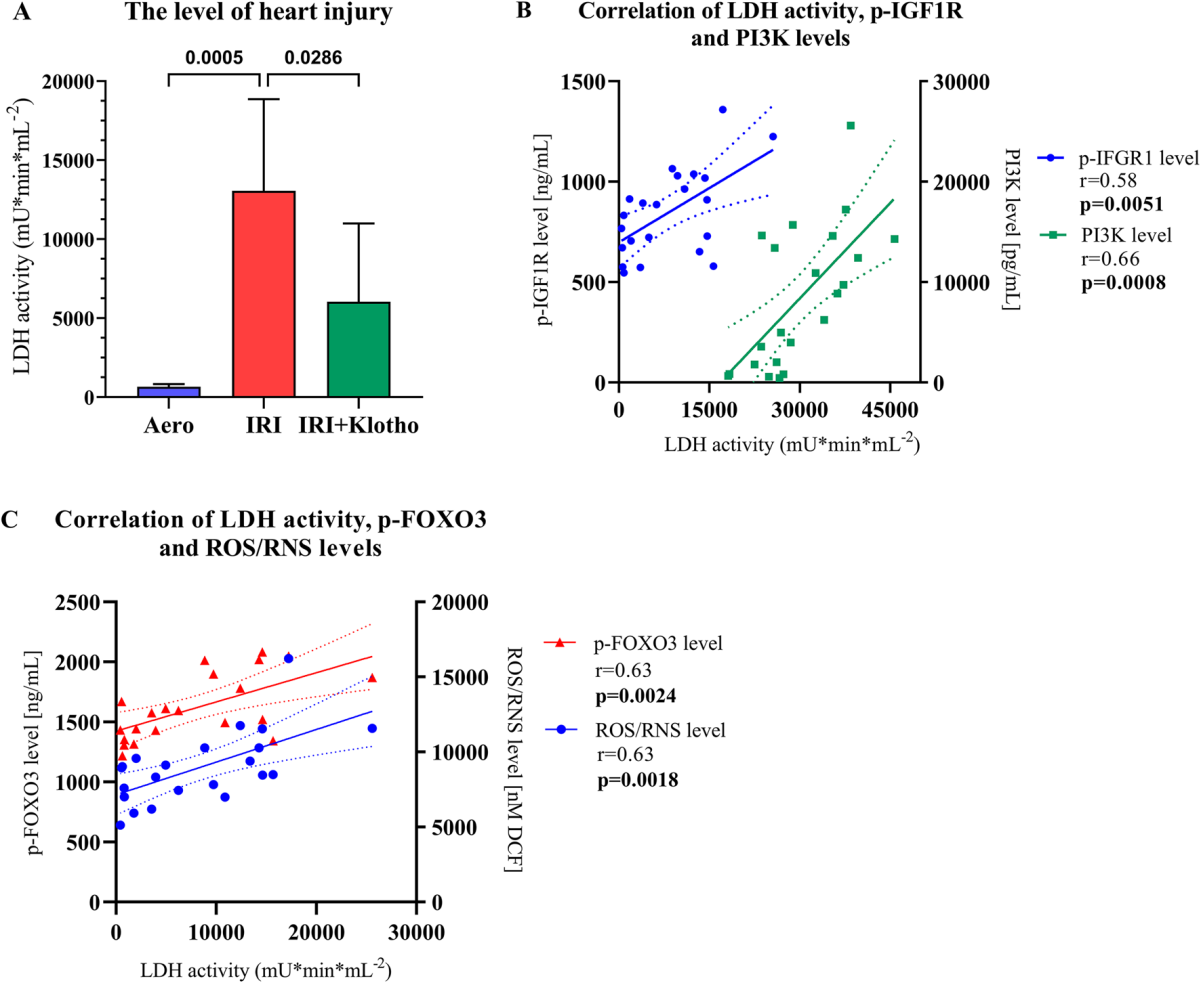
The intensity of heart injury. (**A**) LDH activity in coronary effluents as a marker of cell death; (**B**) Correlation of LDH activity, p-IGF1R and PI3K levels; (**C**) Correlation of LDH activity, p-FOXO3 and ROS/RNS levels; DCF—dichlorodihydrofluorescein; LDH—lactate dehydrogenase; mU—milli international enzyme units; p-FOXO3—phosphorylated forkhead box protein O 3; PI3K—phosphoinositide-3-kinase; p-IGF1R—phosphorylated insulin-like growth factor 1 receptor; ROS/RNS—reactive oxygen/nitrogen species; n_aero_ = 5; n_IRI_ = 11; n_IRI+Klotho_ = 6; mean ± SD.

The LDH activity in coronary effluents was significantly increased in the IRI group as compared to the aerobic control (Fig. 8A). Administration of Klotho protein during IRI significantly reduced damage to the heart (Fig. 8A). The level of heart injury positively correlated with p-IGF1R level (r = 0.58, *p* = 0.0051) (Fig. 8B), PI3K level (r = 0.66; *p* = 0.0008) (Fig. 8B), inactivated FOXO3 level (r = 0.63; *p* = 0.0024) (Fig. 8C) and with the level of oxidative stress (r = 0.63; *p* = 0.0018) (Fig. 8C).

## Discussion

**Figure 9:**
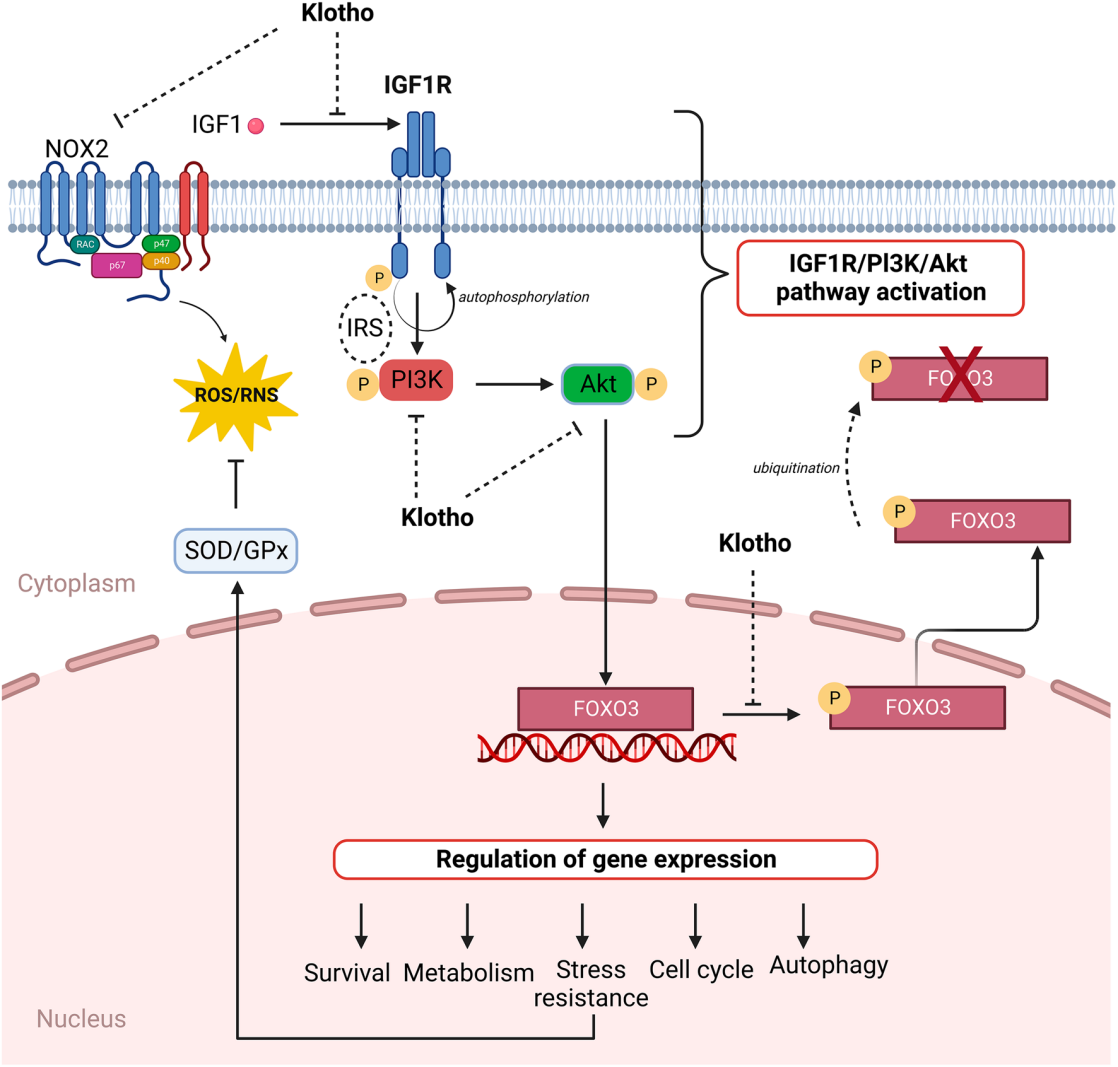
The potential influence of Klotho on the factors in IGF1R/PI3K/AKT signalling pathway and/or oxidative stress during heart IRI. Akt—protein kinase B; FOXO3 –forkhead box protein O 3; GP_X_—glutathione peroxidase; IGF1—insulin-like growth factor 1; IGF1R—insulin-like growth factor 1 receptor; IRS—intracellular adaptor protein insulin receptor substrate; NOX2—nicotinamide adenine dinucleotide phosphate oxidase 2; P—phosphorylation; PI3K—phosphoinositide-3-kinase; ROS/RNS—reactive oxygen/nitrogen species; SOD—superoxide dismutase. Figure created with BioRender (https://biorender.com/).

The leading proponents of myocardial IRI are oxidative stress, pH and calcium paradox, inflammation, metabolic disorders, autophagy and apoptosis^14^. Numerous studies on myocardial injury have demonstrated that during IRI, ion accumulation, damage to the mitochondrial membrane, formation of ROS, disturbances in NO metabolism, and endothelial dysfunction are observed^14^. It is known that mitochondria, and enzymes like xanthine oxidase and NOX are the most important contributors to the exposure of cells to oxidants in IRI^14^. However, it is not fully understood which molecular mechanisms are fundamental in myocardial IRI yet. A combination of additional or synergistic multi-target treatments, and searching for new potential factors are required for optimal cardioprotection. In 1997 Kuro et al. reported an “aging suppressor” gene that encoded a transmembrane protein named Klotho^15^. Disrupted secretion of Klotho protein expedited aging, whereas its high expression extended lifespan in mice^16^. The analysis from the last decade showed that upregulated expression of Klotho is emerging as a promising therapeutic strategy for chronic kidney disease (CKD), acute kidney injury (AKI), and for cardiac hypertrophy, fibrosis and dysfunction^17–19^. FGF23-Klotho axis was proposed as predictive factor of fractures in type 2 diabetics with early CKD^20^. Low Klotho level was associated with mortality and cardiovascular events in haemodialysis patients^21^. For this reason, Klotho can be considered a potential protective factor in cardiovascular diseases as well. The present study confirmed that exogenous Klotho protein contributed to reduction of oxidative stress and damage in hearts subjected to IRI. This effect may be associated with the impact on the IGF1R/PI3K/AKT signalling pathway and increased antioxidant expression. The hypothesis of the influence of Klotho on the different pathway factors has been illustrated in Fig. 9. Furthermore, it is not clear if Klotho acts cardiopreventive and/or therapeutic. Thus, this study focused on the peri-infarction period, where Klotho protein was administered shortly before and after global ischemia. This is the first step of evaluation of the intracellular pathways related to oxidative stress and heart injury, and could be the basis for the next, more specific analysis in the future.

IGF1 has pleiotropic actions in the heart. It plays a role in heart contractility, metabolism, hypertrophy, autophagy, senescence, and apoptosis. The binding of IGF1 to its receptor in the cardiomyocytes, plasma membrane IGF1R, leads to receptor autophosphorylation and activation of a complex signalling cascade (Fig. 9)^22^. It is known that IGF1R can activate two canonical pathways—PI3K/AKT pathway and the extracellular signal-regulated kinase (ERK) pathway^22^. As a result of IGF1R activation, phosphorylation of an intracellular adaptor protein insulin receptor substrate (IRS) occurs. Then, IRS recruits and phosphorylates PI3K, which is followed by AKT phosphorylation (Fig. 9)^23^. In our study, phosphorylation and activation of IGF1R and increase in PI3K level due to IRI was observed. The level of PI3K correlated with phosphorylation of IGF1R, which confirms activation of the pathway during IRI. It was shown that dysregulation of IGF signalling played a role in several kidney diseases, such as proteinuric CKD and polycystic kidneys^24^. Downregulating the IGF1R/PI3K pathway limited loss of podocytes in mice model of diabetic nephropathy^25^. Then, suppression of IGF1R signalling reduced cell death and inflammation, and protected against AKI in mice^26^. Inhibition of the PI3K/AKT/mammalian target of rapamycin (mTOR) pathway reduced oxidative stress and apoptosis, and enhanced autophagy during rat AKI^27^. Considering heart diseases, increased ROS-mediated PI3K/AKT signalling in lipopolysaccharide-induced endotoxemic in vitro and in vivo myocardial injury was shown^28^. It was reported that an underlying mechanism in induced cardiotoxicity was the PI3K/AKT/mTOR pathway. Inhibition of this axis improved cardiac dysfunction, and reduced inflammation, histopathological changes and myocardial apoptosis^29^. PI3K/AKT/mTOR signalling was also related to cardiac fibrosis in mouse ischemia-induced heart failure^30^. Similarly, increased cardiomyocyte hypertrophy through the activation of the PI3K/AKT/mTOR pathway in rat chronic intermittent hypoxia model was shown^31^. Limited activation of PI3K/AKT axis reduced cardiac hypertrophy^32^.

The influence of Klotho on protective mechanisms was well-studied in several kidney diseases. It was reported that Klotho restrained IGF signalling, which protected against renal hypertrophy in diabetic mice or cystic expansion in mice with polycystic kidney disease^33,34^. Reduced nephrotoxicity via negative regulation of the PI3K/AKT pathway by Klotho was shown as well^6^. Scientists reported that the up-regulation of Klotho expression decreased AKT phosphorylation, followed by improved aging-related memory deficits and oxidative stress in mice^35^. Similarly, Klotho was related to IGF1 signalling inhibition, thus to neuroprotection in Alzheimer’s disease mouse model^36^. In human umbilical vein endothelial cells, Klotho ameliorated oxidative stress by regulating the PI3K/AKT/endothelial nitric oxide synthase (eNOS) pathway^37^. IGF/PI3K downregulation was related to antiaging and anticancer activity of Klotho^38,39^. The present results demonstrated that Klotho protein significantly contributed to inhibition of the IGF1R/PI3K/AKT signalling during heart IRI. A similar downregulation of the insulin/IGF1/PI3K/AKT signalling cascade by Klotho and reduction of oxidative stress was observed in HeLa cells^40^. Data showed that IGF1 deficiency and Klotho may reduce the cardiomyocytes’ sensitivity to aging-induced mechanical dysfunction^41^. Importantly, it was established that Klotho is involved in type 2 diabetes and insulin resistance. The anti-aging feature of Klotho was related to inhibition of insulin and IGF1 signalling^42^. Klotho induced insulin resistance in adipocytes by regulation of glucose transporter type 4 and intracellular insulin signalling through AKT^43^. This could explain the influence of Klotho on IGF signalling. Considering the role of IGF1 in cardiac disorders, the ability of Klotho to inhibit IGF in several heart hypertrophy models was investigated. Then, Klotho downregulated IGF/PI3K-dependent calcium channels in the mouse heart, thus protected against cardiac hypertrophy and remodelling^44^. Similarly, increased level of Klotho and inhibition of insulin/IGF1/AKT axis was related to abolished myocardial hypertrophy and fibrosis in mice^45^. It was reported that the level of circulating Klotho was increased in coronary artery disease (CAD) aerobically treated patients compared to non-treated group, but the level of IGF1 was lower^46^. Afterward, Klotho was proposed as a negative regulator for IGF1 and exercise-induced cardiac hypertrophy in women^47^. However, long-lasting chronic resistive training in young adults was related to increased level of IGF1 and heart hypertrophy, but did not influence the level of circulating Klotho^48^.

There are four FOXO family members in mammals: FOXO1, FOXO3, FOXO4 and FOXO6. FOXO3 is predominantly expressed in the heart, brain, kidneys and ovaries^49^. FOXOs regulate their target genes related to metabolism, apoptosis, and cell cycle progression^50^. The association of IGFR/PI3K/AKT and FOXOs was studied in several cell lines and animal models. It was shown that activation of IGFR/PI3K/AKT pathway by IGF1 resulted in phosphorylation of FOXO1/3 in cardiac stem cells, myotubes or cancer cells^50–52^. As a result, translocation from the nucleus to the cytoplasm, and inactivation of FOXO are observed. The phosphorylation of FOXO1/3 by AKT impairs its transcriptional regulation function and triggers its degradation by the ubiquitin–proteasome system (Fig. 9). It was found that inhibition of PI3K/AKT axis could induce the translocation of FOXO1/3 between cytoplasm and nucleus, and its activation^8,49,53^. Interestingly, FOXO1/3 triggered AKT phosphorylation in cardiac myocytes. Then, FOXO-activated AKT feedback inhibition of FOXO was observed^54^. The current study confirmed the phosphorylation of FOXO3 in rat hearts during IRI, leading to its degradation. Inactivation of FOXO3 positively correlated with activation of IGF1R/PI3K signalling pathway. Likewise, Hu et al. (2022) observed low level of FOXO3 due to prolonged myocardial ischemia (MI) in rats^55^. Similar to our results, FOXO proteins were negatively regulated by the IGF1R/PI3K/AKT signalling cascade in breast cancer cells, human brain microvascular endothelial cell IRI, rat spontaneous intracerebral haemorrhage, or mouse cerebral IRI models^56–59^. Inhibition of FOXO3 was related to cardiac hypertrophy and heart failure in mice^60^. Then, downregulation of FOXO1 or FOXO3 expression in mouse heart reduced cardiac function, increased scar formation, enhanced stress-responsive signalling, and strengthened apoptotic cell death after MI^61^. Low expression of FOXO3 was observed also in retinal, hepatic or renal IRI in rats, and in patients with ovarian cancer^62–64^. Importantly, FOXOs were proposed as new therapeutic targets for cardiac diseases^53^. In this study, perfusion of the hearts with Klotho protein significantly inhibited inactivation of FOXO3 during IRI, showing reduced injury and cardioprotection. Interestingly, Frad et al. (2021) reported positive correlation between expression of Klotho and FOXO1 genes in patients with CAD. It was also shown that activation of FOXO1/3 resulted in upregulation of antioxidants and downregulation of proapoptotic molecules, and thus protected against myocardial or cerebral IRI^58,59,65,66^. Then, increased FOXO1/3 expression in the cardiomyocytes inhibited oxidative stress and cell death caused by IRI^61^. These observations were consistent with our results, where Klotho administration inhibited FOXO3 phosphorylation and its subsequent ubiquitination and degradation. An anti-hypertrophic role of FOXO3 in the heart was also shown^67^. The inhibition of cardiomyocyte hypertrophy by FOXO3 was due to enhancing of antioxidant genes and subsequently reduction of ROS levels^68^.

Heart failure is related to oxidative stress, when the production of ROS exceeds the capacity of antioxidant defence. Dependent upon the severity, depletion of endogenous antioxidants in IRI heart is observed^1^. It is known that the FOXOs target genes are related to antioxidative defence. FOXO3 activates the transcription of many antioxidant enzymes, including GP_x_, MnSOD, peroxiredoxin or catalase (Fig. 9)^49^. In this report, the production of antioxidants was negatively correlated with inactivation of FOXO3. Lim et al. (2019) reported decreased expression of MnSOD in tacrolimus (Tac)-induced renal injury mouse model, which was accompanied by increase in PI3K/pAKT levels and inactivation of FOXO3. Then, activated PI3K/AKT factors, FOXO3 phosphorylation, and decreased MnSOD expression due to Tac treatment in the human kidney 2 (HK-2) proximal tubule cell line were observed^69^. The expression of genes involved in oxidative stress resistance was related to FOXO3 activity in neural stem cells^70^. Moreover, FOXO1 upregulated expression of antioxidant enzymes and protected pancreatic β cells against oxidative stress^71^. This study reported that Klotho played a role in the enhancement of SOD and GP_x_ levels in IRI hearts. Similar to the results previously shown, Klotho contributed to PI3K/AKT/FOXO3-related increase in MnSOD expression in Tac-injured HK-2 cells and mouse kidneys or rat renal IRI models^69,72^. Importantly, Ramez et al. (2020) showed that an increase in plasma and myocardial levels of Klotho may boost antioxidant defence during heart IRI^73^. Klotho-enhanced MnSOD and catalase expression were observed in the senescence-accelerated mouse (SAMP8) brain as well. The antioxidative effect of Klotho was related to the inhibition of AKT/FOXO1 pathway^35^. Contrary, aging-related Klotho deficiency was associated with FOXO1 inactivation and downregulation of antioxidant enzymes in aged SAMP8 mice^35^. Upregulating endogenous Klotho expression resulted in the inhibition of IGF1R/AKT pathway, thus enhancement in FOXO1 and antioxidants expression in the 293 T cell line^36^. Klotho was also proposed as a novel therapeutic target for cerebral ischemia, due to its antioxidative effect through AKT/FOXO1 pathway^59^.

The mitochondrial electron transport chain, oxidoreductase, NOX, cytochrome P450 oxidases, and uncoupled NOS are the major enzymatic sources of cellular ROS. NOXs include a group of membrane-bound enzymes and are active in the extracellular space. It is known that during IRI, NOX can produce O_2_^·-^ and other ROS that triggers oxidative stress (Fig. 9)^1^. NOX2 is expressed in the phagocytes, however its production was noted also in many other cell types, e.g., in the cardiomyocytes or endothelial cells^74^. NOX2 is mainly responsible for synthesis of O_2_^·−^, and its increased expression in human cardiomyocytes after acute myocardial infarction was shown^75^. There are numerous studies in cell lines and animal models confirming the impact of oxidative stress during IRI in many organs^59,63,64,72–74^. We have previously reported higher level of NOX2 in human cardiomyocytes injured by IRI, which was accompanied by increased ROS/RNS production^74^. It was indicated that NOX4 activity is regulated via phosphorylation by several kinases, including involvement of IGF1/PI3K/AKT signalling^76–78^. In this study, the amount of NOX2 was positively correlated with p-IGFR1 and PI3K levels, and with FOXO3 inactivation, which shows the axis between IGF1R, NOX, and oxidative stress. IRI led to significantly increased production of total ROS/RNS, including H_2_O_2_ synthesis, which resulted in heart injury. The induction of oxidative stress correlated with activation of IGFR1/PI3K pathway and inactivation of FOXO3. There was also a positive correlation between ROS/RNS and NOX2 levels, which confirms the influence of NOX2 on oxidants formation. Finally, impaired IGF1R/PI3K/FOXO3 signalling and oxidative stress resulted in heart injury. Administration of Klotho protein significantly reduced NOX2 production, and then the oxidative stress and injury in IRI hearts. These results are consistent with our previous observations, where Klotho contributed to the reduction in NOX2/4 level, protected from oxidative stress, and enhanced total antioxidant capacity in the cardiomyocytes subjected to IRI^74^. Similarly, Klotho ameliorated oxidative stress caused by IRI in mouse kidneys and renal tubular epithelial cells^79^. Klotho efficiently suppressed NOX2/4 and ROS overproduction in hypertrophic neonatal rat cardiomyocytes and heart tissue in CKD-associated left ventricular hypertrophy mouse model^80^. An increase in NOX1, 2 and 4 levels, and induced oxidative stress were also found in calcified vascular smooth muscle cells that were mitigated after administration of exogenous Klotho. Afterward, NOX2-derived ROS production in the aorta of vascular calcification rats was reduced after Klotho injection^81^. There are also several reports confirming the antioxidative role of Klotho, where Klotho targeted oxidative stress during renal or brain injury^6,35,59,69^. Our research has supplemented the knowledge about the antioxidative role of Klotho also in heart injury.

## Conclusions

This study demonstrates that Klotho protein contributed to the IGF1R/PI3K/AKT signalling pathway inhibition during IRI of the rat heart. Both, IGF1R, and subsequent PI3K/AKT downregulation significantly counteracted IRI-induced NOX2 activity, oxidative stress and injury that followed. Klotho influenced FOXO3 activity, thus increasing antioxidative capacity in the heart tissue. To the best of our knowledge, this is the first study demonstrating that Klotho may protect against oxidative stress via inhibition of the IGF1R/PI3K/AKT pathway in the heart IRI model. Summing up, Klotho can be recognized as a novel cardiopreventive/cardioprotective agent.

## Data Availability

The data that support the findings of this study are available from the corresponding author upon reasonable request.
